# Ruptured Aortic Arch Aneurysm Without Surgical Intervention Resulting in 11-Day Survival in a Woman: An Autopsy-Proven Aortoesophageal Fistula

**DOI:** 10.7759/cureus.90950

**Published:** 2025-08-25

**Authors:** Takuya Kubota, Shintaro Koizumi, Masahiro Higashi, Kiyoshi Mori, Hirotsugu Iwatani

**Affiliations:** 1 Department of Nephrology, National Hospital Organization Osaka National Hospital, Osaka, JPN; 2 Department of Nephrology, Osaka University Graduate School of Medicine, Suita, JPN; 3 Department of Radiology, National Hospital Organization Osaka National Hospital, Osaka, JPN; 4 Department of Central Laboratory and Surgical Pathology, National Hospital Organization Osaka National Hospital, Osaka, JPN

**Keywords:** aortic aneurysm, aortoesophageal fistula, atherosclerosis, autopsy, hemodialysis, rupture

## Abstract

In general, rupture of an aortic aneurysm causes immediate death and seldom sustains the patient’s life without surgical intervention. Here, we report a rare case of a 79-year-old woman on maintenance hemodialysis with a ruptured aortic arch aneurysm who survived more than one week without persistent shock status and surgical intervention. She was transferred to our hospital with her complaints of fever, dyspnea, and chest oppression. Laboratory data showed elevated inflammatory reaction and increased fibrinolysis. On her examination, plain computed tomography (CT) showed thickening of the esophageal wall and mediastinal mass shadow, implying esophageal cancer with its tracheal invasion and its lymph node metastasis in addition to pneumonia. Gastrointestinal fiberscope (GIF) on the fourth day showed extrinsic compression of the esophagus, and the patient complained of augmented chest pain immediately after starting hemodialysis. The repeated plain CT showed an enlarged mediastinal mass, and contrast-enhanced CT showed findings of a ruptured aortic arch aneurysm. She and her family did not want to undergo a surgical operation due to the high risk of intraoperative death, and the maintenance hemodialysis was discontinued for fear of relapse or rupture. Melena was observed from the 10th day, and she finally died on the 11th day. The autopsy revealed marked atherosclerosis of the aorta, a 35 mm diameter aortic aneurysm in the aortic arch, and aortoesophageal fistula. Even in cases of chest pain without immediate death or sustained shock status, a rupture of an aortic aneurysm should be considered for the differential diagnosis.

## Introduction

Aortic aneurysms are defined as “the circumferential or local enlargement (in diameter) or thickening of a part of the entire circumference of the aortic wall” [1]. Aneurysms are classified into thoracic, thoracoabdominal, and abdominal aneurysms by location. Thoracic aortic aneurysms (TAAs) are often caused by atherosclerosis, hypertension, or chronic conditions like renal failure, which accelerate vascular degeneration. In addition, patients on hemodialysis face increased TAA risk due to chronic inflammation and vascular calcification. It is previously reported that TAAs occur in six to 10 per 100,000 person-years [2], and the rupture is one of the life-threatening complications. The majority of patients with aortic rupture continuous bleeding cannot survive even with massive transfusion and large doses of catecholamine [3]. In addition to pain-relieving and blood pressure-lowering, surgical operations are highly important to prevent immediate death. In fact, the aortic perforation to the pericardial sac (heart’s protective layer), mediastinum (chest cavity between lungs), or thoracic cavity is fatal, and it is almost the case that the patient dies immediately after the onset, before the appearance of the rescuer [1]. Furthermore, the rupture may lead to catastrophic bleeding or fistulae with adjacent structures, such as the esophagus, as seen in this case. Thus, it is natural that there are few reports showing the survival after the onset of rupture of TAA without surgical operation [4,5]. Here, we report a 79-year-old woman with a ruptured aortic arch aneurysm on maintenance hemodialysis, who survived more than one week without surgical intervention and whose autopsy revealed aortoesophageal fistula.

## Case presentation

A 79-year-old woman was transferred and admitted to our hospital due to fever, dyspnea, and chest oppression. She had a past history of hypertension, diabetes mellitus, and dyslipidemia and had been on maintenance hemodialysis for one year. She also had coronary artery disease, although she had refused to receive percutaneous coronary intervention. She did not have any past history of surgical operation, either.

Her vital signs at the emergency room were as follows: blood pressure 119/50 mmHg, pulse rate 94 beats per minute, and body temperature 39.0 degrees Celsius. Of note, she was not in shock status. On physical examination, she complained of pain around the left brachium and left scapula, and below the left scapula, while cardiac and respiratory sounds were normal. Her laboratory data showed the elevated inflammatory reaction (white blood cells (WBC) 14400/μL and C-reactive protein (CRP) 30.99 mg/dL) and increased fibrinolysis (D-dimer 8.12 μg/mL) (Table 1).

**Table 1:** Laboratory data on the day of admission Laboratory data on the day of admission (day 1) showed a high inflammatory reaction and a generalized fibrinolytic state. Rupture of an aortic aneurysm contributed to the elevation of inflammatory markers and D-dimer levels. Hemoglobin level was within normal range despite the history of a ruptured aortic arch aneurysm.

Laboratory test	Patient value (day 1)	Patient value (day 4)	Reference range
Complete blood count			
White blood cells (/μL)	14400	12400	3300-8600
Red blood cells (/μL)	3.78×10^6^	3.42×10^6^	3.86×10^6^-4.92×10^6^
Hemoglobin (g/dL)	12.3	11.0	11.6-14.8
Hematocrit (%)	37.8	34.6	35.1-48.2
Platelets (/μL)	37.1×10^4^	36.2×10^4^	15.8×10^4^-34.2×10^4^
Blood chemistry			
Aspartate transaminase (U/L)	40	16	13-30
Alanine transaminase (U/L)	32	20	7-23
Total protein (g/dL)	6.1	5.7	6.6-8.1
Albumin (g/dL)	2.8	2.5	4.1-5.1
C-reactive protein (mg/dL)	30.99	18.91	0.00-0.14
Urea nitrogen (mg/dL)	23	45	8-20
Creatinine (mg/dL)	4.31	7.18	0.46-0.79
Sodium (mEq/L)	138	134	138-145
Chloride (mEq/L)	99	96	101-108
Calcium (mg/dL)	8.3	8.1	8.8-10.1
Phosphate (mg/dL)	4.4	5.9	2.7-4.6
Troponin I (pg/mL)	52	43.9	0.0-26.2
Blood coagulation test			
D-dimer (μg/mL)	8.12	9.58	0.00-1.00

**Figure 1:**
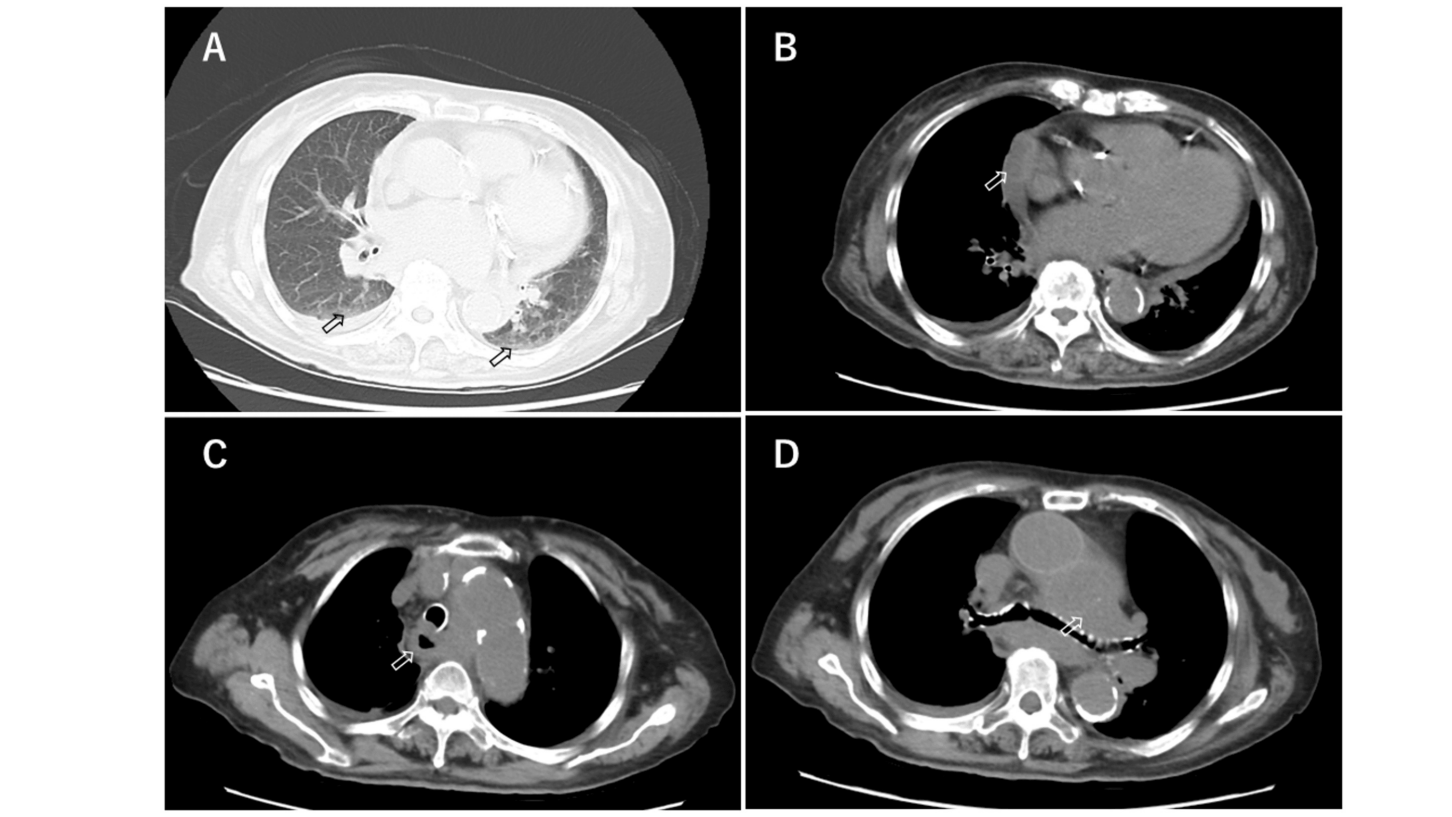
Chest plain computed tomography (CT) on the day of admission Chest plain CT on the day of admission when the patient manifested with fever, dyspnea, and chest oppression/pain showed ground glass opacity in the lingular segment of both lungs and dorsal inferior lobe (A, arrow), soft pericardial shadows (B, arrow), thickening of the esophageal wall (C, arrow), and mediastinal mass shadow (D, arrow). These findings raised a suspicion of esophageal cancer with its tracheal invasion and its lymph node metastasis in addition to pneumonia, initially. CT slice thickness: 5 mm.

Chest plain CT showed ground glass opacity in the lingular segments of both lungs, dorsal inferior lobe, soft pericardial shadows, thickening of the esophageal wall, and mediastinal mass shadow, raising a suspicion of esophageal cancer with its tracheal invasion and its lymph node metastasis in addition to pneumonia (Figure 1).

Considering these clinical examinations, the initial differential diagnoses for her included bacterial pneumonia, interstitial pneumonia, opportunistic infections, pericarditis, mediastinitis, amyloidosis, sarcoidosis, and esophageal cancer with lymph node metastasis. The laboratory data showed no elevated Krebs von den Lungen-6 (KL-6) and surfactant protein D (SP-D) levels (279 units/mL, 35.7 ng/mL, respectively). There was also no elevation of β-D-glucan (15.7 pg/mL), and cytomegalovirus antigen and aspergillus antigen were negative. In addition, the blood culture was negative. Sputum examination was not performed because of no expectoration. Thus, active infection was not obviously considered. There existed neither ST-T change in the electrocardiogram nor elevation of creatine kinase-MB level (1 U/mL on day 1). The concentration of troponin I was slightly elevated to 52 pg/mL on day 1, but it showed a decrease to 47.8 pg/mL three hours after admission and a further decrease to 43.9 pg/mL on day 4, as shown in Table 1. Creatine kinase-MB level was also within normal range (1 U/mL) on day 4, and lactate dehydrogenase levels were within normal range both on day 1 (187 U/L) and day 4 (153 U/L). Potassium levels were within normal range both on day 1 (4.1 mEq/L) and day 4 (4.1 mEq/L). In addition, pericardial friction sound was not heard, and transthoracic echocardiography by a cardiologist revealed no sign of pericarditis. The infectious disease screening was performed on day 1, with all results negative (Hepatitis B surface antigen (-), Hepatitis B surface antibody (-), Hepatitis C antibody (-), rapid plasma reagin (-), and Treponema pallidum hemagglutination assay (-)).

Although there existed a slight elevation of lysozyme (22.6 mg/mL), there was no bilateral hilar lymphadenopathy, eye involvement, and no elevation of serum angiotensin converting enzyme, which suggested sarcoidosis was less likely. There were no tracheoesophageal fistulae and invasive pneumonitis; mediastinitis was less likely.

A couple of hours after admission, her chest pain spontaneously disappeared. We defined the day of admission as day 1. On day 4, several tumor markers were measured, including squamous cell carcinoma antigen (SCC), soluble interleukin-2 receptor, and progastrin-releasing peptide (proGRP) (Table 2), which were not considered to be significantly elevated in a patient with end-stage renal failure.

**Table 2:** Laboratory test for tumor markers on day 4 Laboratory test for tumor markers on day 4 showed a slight elevation of squamous cell carcinoma antigen (SCC) and progastrin-releasing peptide. This SCC elevation was not significant due to the condition of renal failure. Soluble interleukin-2 receptor was high, but end-stage renal failure and high CRP value made it difficult for us to consider it a meaningful elevation. Neuron-specific enolase and progastrin-releasing peptide were tested for suspected lung neuroendocrine tumor and lung small cell carcinoma, respectively. Sialylated Lewis X antigen, cytokeratin 19 fragment, and soluble interleukin-2 receptor were tested for suspected adenocarcinoma, squamous cell carcinoma, and lymphoma, respectively.

Laboratory test	Patient value	Reference range
Squamous cell carcinoma antigen (ng/mL)	2.3	0.6-1.5
Carcinoembryonic antigen (ng/mL)	2.2	0.0-4.0
Neuron-specific enolase (ng/mL)	10.6	0.0-16.3
Sialylated Lewis X antigen (unit/mL)	31	0-38
Progastrin-releasing peptide (pg/mL)	191	0-181
Cytokeratin 19 fragment (ng/mL)	2.2	0.0-2.8
Soluble interleukin-2 receptor (unit/mL)	1590	121-613

**Figure 2:**
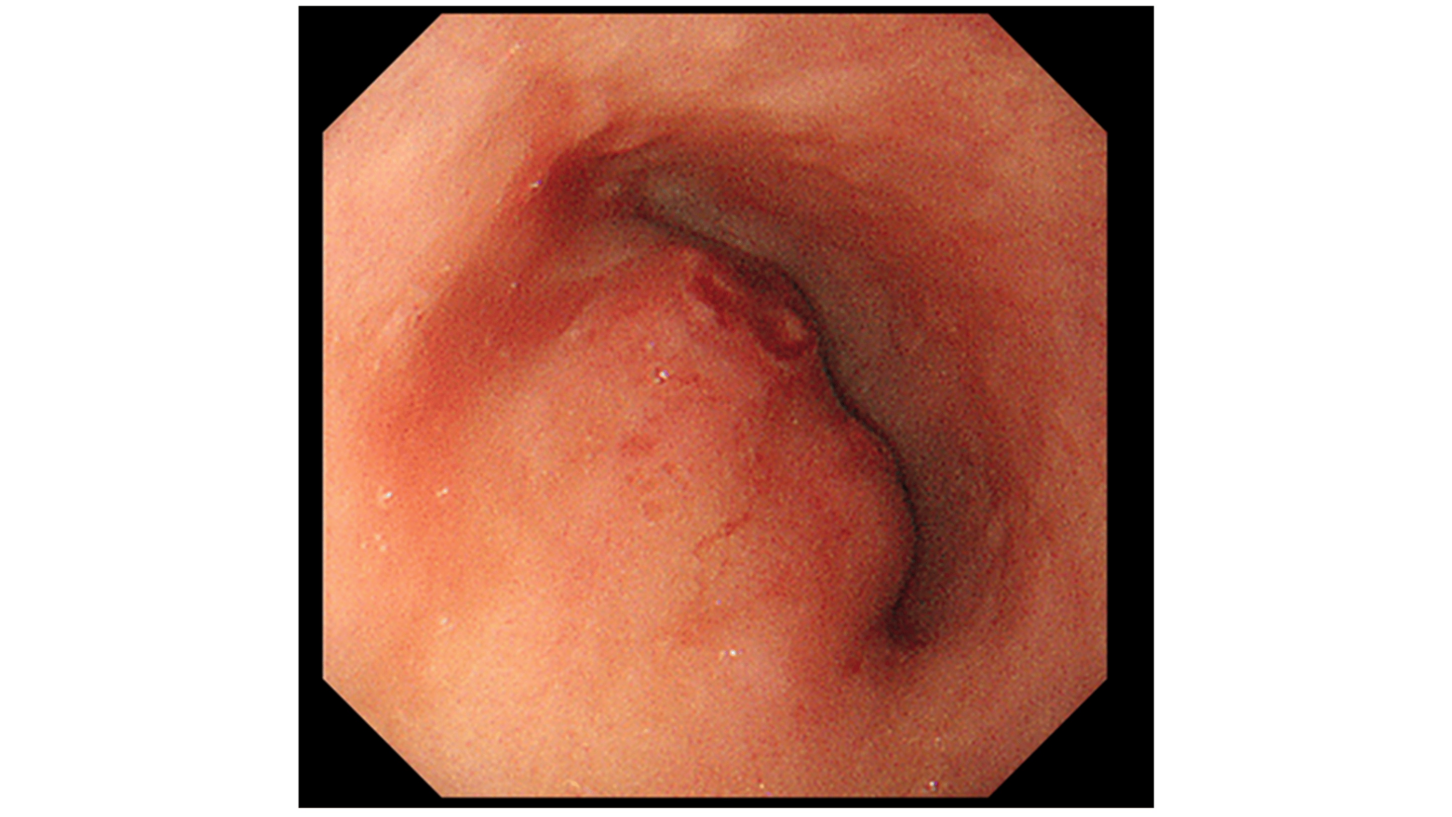
Gastrointestinal fiberscope performed on day 4 An extrinsic compression of the esophagus was found by a gastrointestinal fiberscope performed on day 4. There was no malignant finding. There was a pulsation in the compression area, which turned out to be a later aortic arch aneurysm.

On the same day, a gastrointestinal fiberscope (GIF) was performed, which showed no malignant findings, but extrinsic compression of the esophagus with pulsation (Figure 2).

**Figure 3:**
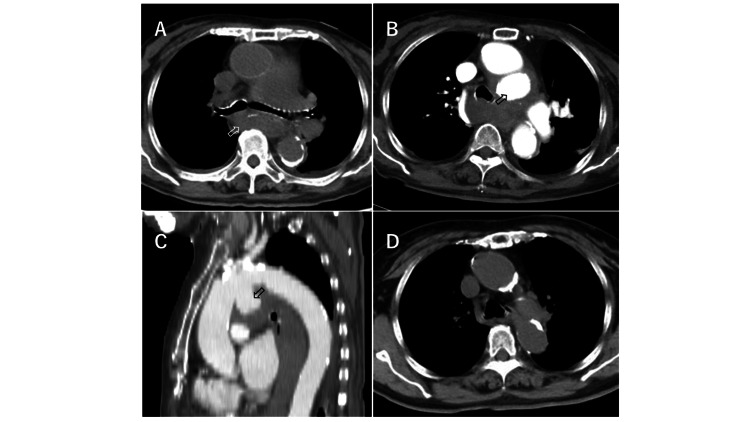
Chest CT after hemodialysis on day 4 Chest plain CT after hemodialysis on day 4, when the patient presented with relapsing severe chest pain, indicated an enlarged mediastinal mass (A; arrow), which turned out to be a hematoma due to the rupture of an aortic arch aneurysm. Finally, the diagnosis of aortic arch rupture was confirmed by contrast-enhanced CT (B, C, arrow, contrast dose: 100 mL). In fact, there was no aortic aneurysm 20 months before the admission (D). CT slice thickness: 5 mm.

After the GIF, the patient received hemodialysis. Immediately after the start of dialysis using heparin (1000 units bolus, 500 units per hour infusion), the patient presented with severe chest pain again, and she was referred for CT imaging. There was an enlarged mediastinal mass shadow on plain CT (Figure 3A), and contrast-enhanced CT (Figures 3B, 3C) showed an aortic arch aneurysm and an enlarged hematoma in the mediastinum, which was not observed 20 months before the admission and leading to the diagnosis of a rupture of aortic arch aneurysm (Figure 3D).

**Figure 4:**
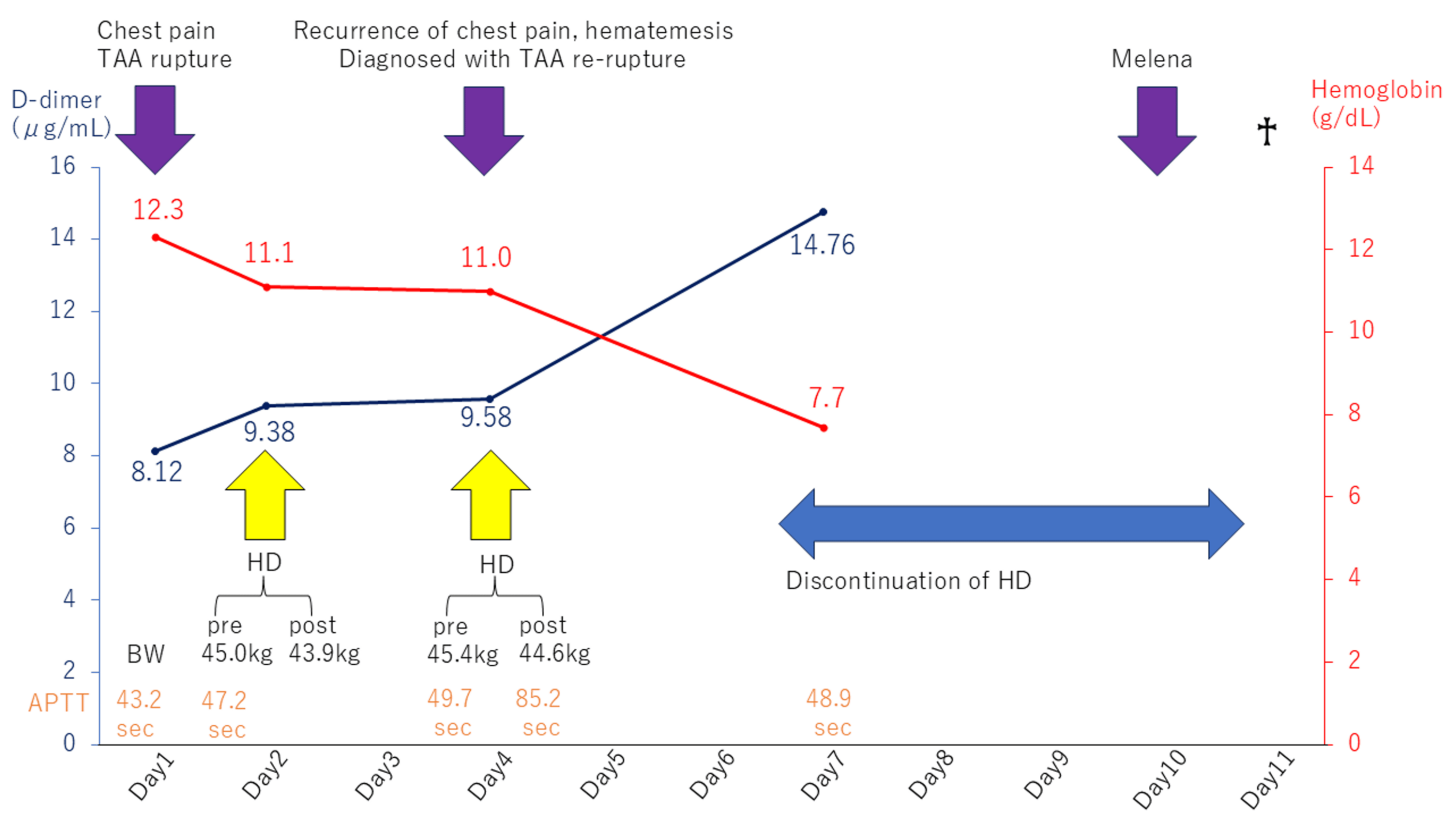
Clinical course There was a continuous elevation of D-dimer levels during the clinical course. Simultaneously, a worsening of anemia was also observed, which was compatible with the occurrence of aneurysm rupture. The data of activated partial thromboplastin time (APTT) (seconds) in orange are shown in the lowest part of the chart. The data of body weight (BW) (kilograms) in black are shown in the lowest part but one. HD: hemodialysis; TAA: thoracic aortic aneurysm

The cardiovascular surgeons recommended surgical operations, but the patient and her family did not wish to undergo a surgical operation due to the high risk of intraoperative death. Owing to the risk of re-rupture of the aortic aneurysm during hemodialysis, maintenance hemodialysis was not performed further. The patient's vital signs remained stable, and there was a temporal elevation of D-dimer level (14.76 μg/mL on day 7) and a decline of hemoglobin level (7.7 g/dL on day 7), following relapsing TAA rupture on day 4 (Figure 4).

**Figure 5:**
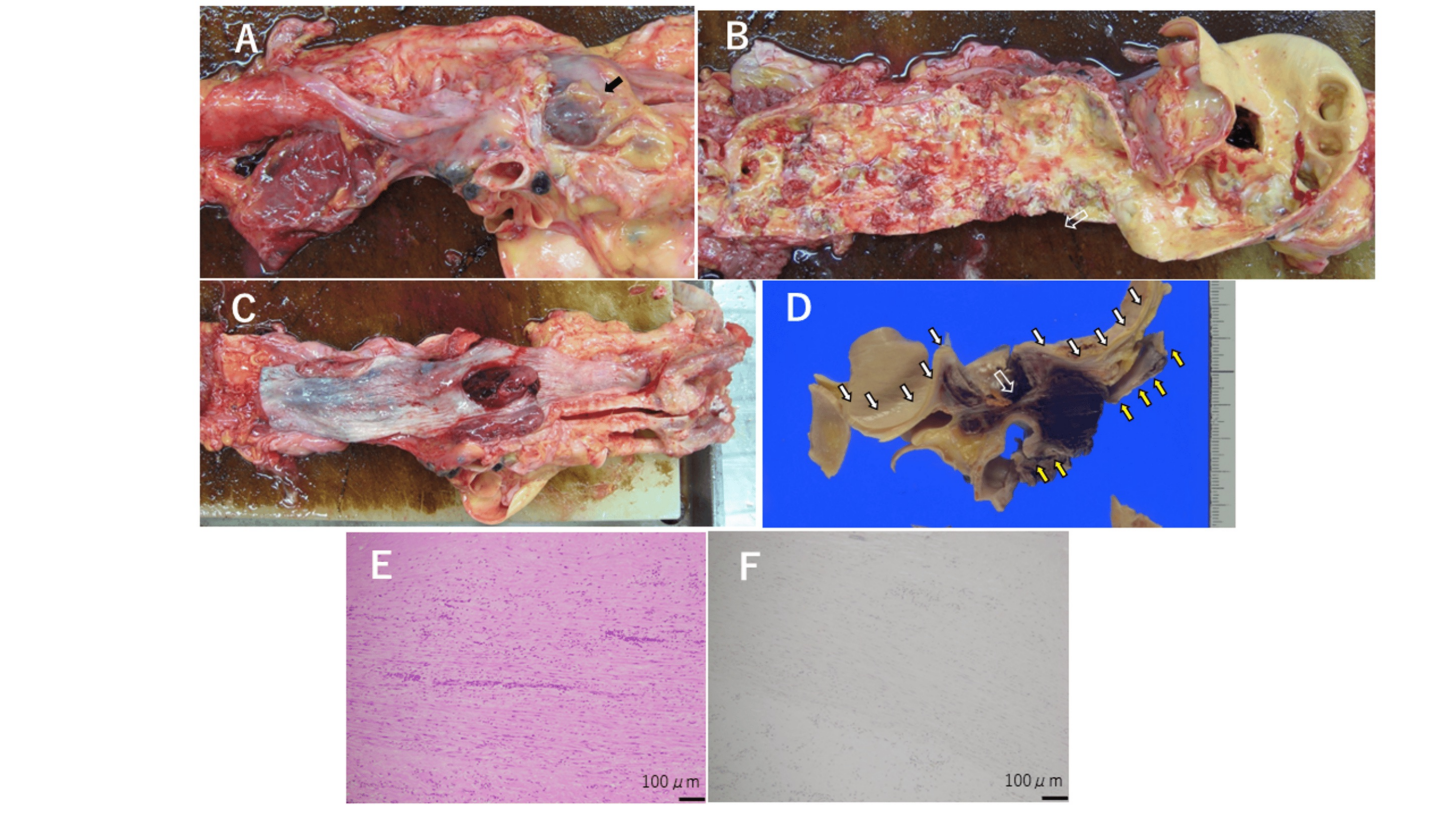
Autopsy Autopsy revealed a 35 mm-diameter aortic aneurysm in the aortic arch (A, arrow), and prominent atherosclerosis of the aorta (B). Venous hemostasis was observed in the esophagus (C). Aortoesophageal fistula also existed, which was also proved pathologically (D, open arrow) (white and yellow solid arrows indicate the aortic intima and the esophageal mucosa, respectively). Hematoxylin and eosin stain (E) and Congo red stain (F) of the aortic wall were performed. The Congo red stain was negative. There were no pathological findings suggestive of malignancy, vasculitis, or amyloidosis.

Surprisingly, her vital signs remained stable on day 10 (blood pressure 137/51 mmHg, pulse rate 79 beats per minute, and body temperature 37.2 degrees Celsius). From day 10, she began to have melena, and she died on day 11. Autopsy revealed marked atherosclerosis of the aorta, a 35 mm-diameter aortic aneurysm in the aortic arch, and aortoesophageal fistula. There were no pathological findings suggestive of malignancy, vasculitis, or amyloidosis as revealed by a negative Congo red stain (Figure 5F).

## Discussion

This is the report of a woman on maintenance hemodialysis who survived a rupture of an aortic arch aneurysm with autopsy-proven aortoesophageal fistula without surgical operation for more than a week, without persistent shock status. The CT findings were not indicative of the ruptured aortic aneurysm due to the sustained blood pressure initially, but the retrospective consideration of the severe pain and the gastrointestinal endoscopic findings of the extrinsic compression of the esophagus with pulsation strongly indicated that the ruptured aortic aneurysm and the subsequent aortoesophageal fistula would have been the main cause of the death revealed by autopsy. Contrast-enhanced CT was not performed on the day of admission because it was considered to be an excessive medical examination since the patient’s vital signs were stable and her chest pain disappeared spontaneously, nor was the further elevation of troponin I level over time. However, this case prompted us to consider that clinicians should not hesitate to perform contrast-enhanced CT scans to rule out the presence of any aortic aneurysm, even in such a situation.

Of all TAAs, aneurysms of the aortic root, ascending aorta, or both are most common (~60%), followed by those of the descending aorta (~30%) and arch (<10%) [6]. The causes of TAAs are classified into atherosclerotic, infected, traumatic, inflammatory, congenital, heritable, etc. [6]. It is reported that the most common cause of the aortic arch aneurysm is the existence of a previous aortic dissection.

Basically, surgical operation for TAAs is usually performed when TAAs are confirmed. Thus, there are a few reports on medication treatments [1]. Most aortic aneurysms are asymptomatic, but TAA may present with hoarseness, shortness of breath [7], dysphagia [6], and vague back pain. Risk factors for TAA include age, hypertension, smoking, dyslipidemia [8], history of ischemic heart disease or chronic renal failure [9], bicuspid valve [10], and genetic disorders such as Marfan syndrome.

The patient in our case presented with dyspnea and chest oppression, which were considered to be the symptoms of TAA rupture. In addition, the patient initially presented with motion pain around the left brachium and left scapula, and below the left scapula, which probably coincided with the site of the ruptured aneurysm, and may also be symptoms of TAA rupture. It could be also speculated that esophageal compression (Figure 1C) by hematoma induced by rupture of TAA might have contributed to chest oppression and pain on day 1, and that relapsing rupture of TAA on day 4 caused the severe chest pain, which coincided with the enlarged hematoma in the mediastinum by CT findings (Figures 3A, 3B). Since there was no past medical history of aortic dissection, trauma, or obvious infection, in addition to no aortic aneurysm 20 months before admission in this case, it is suspected that the risks for this case were old age, hypertension, dyslipidemia, history of ischemic heart disease, and chronic renal failure. Although our patient had end-stage kidney disease requiring hemodialysis for one year, amyloidosis was denied by a negative Congo red stain. Thus, it is natural to consider that the etiology of TAA for this patient would have been atherosclerosis.

In truth, the mortality of TAA rupture is extremely high. In fact, it is previously reported that only 64 in 158 patients with TAA rupture were transferred to emergency room alive albeit 11 (7%) and 83 (53%) patients died in welfare facilities for the elderly or outside the hospital respectively in a two-point observation study conducted in Stockholm, Sweden in the 1980’s [11]. Of these patients, surgery was performed in two cases, and of the 135 cases for which the time course from symptom onset to death could be established, the time to death was reported as zero to six hours in 73 (54%), seven to 24 hours in 30 (22%), and more than 24 hours in 32 (24%) of the patients [11]. The risk of dissection or rupture of an aortic aneurysm is related to aortic diameter and is presumed to be similar in the arch as in other thoracic sites. However, there are no large reports examining arch dimensions alone. In asymptomatic patients with low surgical risk, surgical replacement therapy is reasonable when the diameter is 55 mm or greater [12,13].

There is a report that D-dimer (<0.5 μg/mL) is useful for ruling out acute aortic syndromes, including ruptured aortic aneurysms [14]. There is also a report that the median D-dimer in the whole patients on hemodialysis was 0.966 (inter-quartile range (IQR) 0.524-1.947) μg/mL and D-dimer was positive (>0.50 μg/mL) in 75% of cases, while the median D-dimer in hemodialysis patients without acute illness or predisposing chronic diseases was 0.5385 (IQR 0.359-0.966) μg/mL, positive in 52% of them [15]. In the present case, the diameter of the aneurysm at autopsy was 35 mm, and D-dimer levels were very high considering that the normal upper limit of D-dimer was 1.0 μg/mL as in Table 1 and the third quartile of D-dimer in the whole patients on hemodialysis was 1.947 μg/mL. Moreover, D-dimer showed a steady increase over time after day 1, which was further accelerated after day 4, when the rupture should have occurred (Figure 4). However, the regression analysis did not show that the temporal increase of D-dimer was statistically significant (p=0.05336), probably due to the small number of data points.

In addition, it is also reported that inflammatory reactions were significantly elevated in patients with ruptured aortic aneurysm (median CRP value: 2.4 (IQR 0.65-8.6) mg/dL, median WBC value: 13200 (IQR 10500-17000)/μL) and that the elevation of CRP, WBC, and fibrinogen may be an indicator of acute phase response (i.e., rupture) due to vascular tissue damage and inflammation in atherosclerotic disease [16]. Hence, it is natural to consider that rupture of the aortic aneurysm contributed to the elevation of inflammatory markers in our case. It could be speculated that the massive bleeding from a ruptured aortic aneurysm was absent and that the bleeding may have been hemostatically stopped to contain the ruptured TAA locally by the mediastinal hematoma or possible clot formation. It could also be conceivable that the hemodynamic changes and the use of heparin during hemodialysis may have contributed to the re-rupture of the aortic aneurysm. Indeed, the patient complained of chest pain on days 1 and 4, when hemodialysis was performed. When we focus on the hemodialysis on day 4, she might have been withstanding the hemodynamic pressure induced by the body weight increase from 43.9 kg after hemodialysis on day 1 to 45.4 kg immediately before the hemodialysis. The serial data of activated partial thromboplastin time (APTT) were within the range of 43.2 seconds to 47.2 seconds before the hemodialysis on day 4. Three minutes after the start of hemodialysis on day 4, she suddenly complained of severe chest pain and could not remain in bed. At the start of the hemodialysis, the bolus infusion of 1000 units of heparin was performed, and this heparin probably could have triggered the re-rupture. Moreover, she might have been overhydrated due to discontinuation of hemodialysis, considering her weight gain between hemodialysis was approximately 1.5 kg, although the body weight was not measured after day 5 due to the best supportive care. This possible overhydration status might have resulted in the elevated hydrostatic pressure and further aggravation of an aortoesophageal fistula [17,18], followed by melena, which suggested TAA rupture.

Aortoesophageal fistula, which forms through the aorta and esophagus due to various causes, is a rare but fatal cause of upper gastrointestinal bleeding. The main causes are aneurysms and perforating ulcers of the thoracic aorta, among which the TAA is the most common cause (about 60-75%). The classic clinical triad is reported to be midthoracic pain or dysphagia, a sentinel episode of hematemesis, followed by fatal exsanguination. The period between the sentinel episode of hematemesis and fatal exsanguination is reportedly days long, such as two days or three days [19]. In the report, the sentinel hemorrhage may be due to hemostasis because of a small aortic orifice of the fistula, and the pathological analysis revealed that the intima of the thoracic and abdominal aorta exhibited severe atherosclerotic changes. In that autopsy, thrombus formation was confirmed that covered the opening of the aortic orifice of the fistula. These features are almost consistent with our case, although a clear thrombus was not detected.

The report and our present case strongly suggest that even if rupture of an aortic aneurysm occurs, it sometimes only remains as a sentinel hemorrhage due to hemostasis for days, and finally leads to catastrophic bleeding. Especially, our present case has a long history from the first aortic rupture (day 1), then re-rupture (day 4), then melena (day 10), to death (day 11).

To investigate similar cases, a thorough literature review was performed using the PubMed database. Terms such as "aneurysm," "rupture," "hemodialysis," and "aortoesophageal" were carefully selected. Among patients on hemodialysis, there are only a few reports referring to ruptured aortic arch aneurysm [5,20] and their autopsy findings [20], which do not show any aortoesophageal fistula. Furthermore, there are a few reports showing the survival beyond the acute phase of ruptured TAA without surgical operation [4,5]. Thus, to the best of our knowledge, this is the first report of a ruptured aortic arch aneurysm in a patient on maintenance hemodialysis, who survived more than one week without surgical intervention, and whose autopsy finding revealed aortoesophageal fistula.

## Conclusions

We experienced a rare case of a woman on maintenance hemodialysis who survived more than one week without surgical intervention after a ruptured aortic arch aneurysm. The diagnosis was difficult to make. Aortoesophageal fistula was confirmed by autopsy, and amyloidosis was denied. Even in cases of chest pain without immediate death or sustained shock status, a rupture of an aortic aneurysm should be considered for the differential diagnosis, and contrast-enhanced CT should be promptly performed, especially when esophageal compression, mediastinal widening, or a sudden rise in D-dimer or inflammatory markers exist.
